# Ability of wild type mouse bioassay to detect bovine spongiform encephalopathy (BSE) in the presence of excess scrapie

**DOI:** 10.1186/s40478-015-0194-2

**Published:** 2015-04-03

**Authors:** Erica Corda, Leigh Thorne, Katy E Beck, Richard Lockey, Robert B Green, Christopher M Vickery, Thomas M Holder, Linda A Terry, Marion M Simmons, John Spiropoulos

**Affiliations:** School of Veterinary Medicine, University of Milan, Milan, Italy; Current address: Department of Ecosystem and Public Health, Faculty of Veterinary Medicine, University of Calgary, Calgary, Canada; Animal and Plant Health Agency, Woodham Lane, New Haw, Addlestone Surrey, KT15 3NB UK; Current address: University of Southampton, Southampton, Hampshire SO17 1BJ UK; Current address: Biogen Idec, 14 Cambridge Center, Cambridge, MA 02142 USA

**Keywords:** BSE, Scrapie, Co-infection, Bioassay, Mouse

## Abstract

**Introduction:**

Scrapie and bovine spongiform encephalopathy (BSE) are transmissible spongiform encephalopathies (TSEs) which naturally affect small and large ruminants respectively. However, small ruminants, which are susceptible to BSE under experimental conditions, have been exposed to the same or similar contaminated food additives as cattle. To date two natural cases of BSE in small ruminants have been reported. As a result surveillance projects, combined with appropriate control measures, have been established throughout the European Union (EU) to minimize the overall incidence of small ruminant TSEs. Although BSE can be differentiated from classical scrapie (subsequently referred to as scrapie) if appropriate discriminatory tests are applied, the value of these tests in BSE/scrapie co-infection scenarios has not been evaluated fully. Mouse bioassay is regarded as the gold standard regarding differentiation of distinct TSE strains and has been used as to resolve TSE cases were laboratory tests produced equivocal results. However, the ability of this method to discriminate TSE strains when they co-exist has not been examined systematically. To address this issue we prepared *in vitro* mixtures of ovine BSE and scrapie and used them to challenge RIII, C57BL/6 and VM mice.

**Results:**

Disease phenotype analysis in all three mouse lines indicated that most phenotypic parameters (attack rates, incubation periods, lesion profiles and Western blots) were compatible with scrapie phenotypes as were immunohistochemistry (IHC) data from RIII and C57BL/6 mice. However, in VM mice that were challenged with BSE/scrapie mixtures a single BSE-associated IHC feature was identified, indicating the existence of BSE in animals where the scrapie phenotype was dominant.

**Conclusions:**

We conclude that wild type mouse bioassay is of limited value in detecting BSE in the presence of scrapie particularly if the latter is in relative excess.

**Electronic supplementary material:**

The online version of this article (doi:10.1186/s40478-015-0194-2) contains supplementary material, which is available to authorized users.

## Introduction

Classical scrapie (subsequently referred to as scrapie) and Bovine Spongiform Encephalopathy (BSE) are transmissible spongiform encephalopathies (TSEs), a group of fatal neurodegenerative disorders of animals and man which are characterised by the deposition of a misfolded isoform (PrP^Sc^ or *prion*) of a cellular protein (PrP^C^), spongiosis and gliosis. Even though scrapie has been endemic in sheep and goats for centuries it is believed not to have represented a risk for human health [1,2]. On the contrary, BSE is a relatively new prion disease [3], and has been linked with variant Creutzfeldt-Jakob disease (vCJD) a form of CJD which usually affects young subjects [4-6].

The origin of BSE is still unclear. Two main theories suggest either a sporadic cattle disease or a cattle-adapted form of scrapie that crossed the species barrier [7,8]. The recycling of ruminant tissue into meat and bone meal, which was commonly fed as an ingredient of concentrated feed supplements, in combination with changes in rendering procedures that were implemented in the UK in the 1980s, exposed not only cattle but also small ruminants to infectious prions [8,9]. Furthermore, experimental challenge of small ruminants with BSE proved that they are susceptible, resulting in a disease with clinical signs that are indistinguishable from those caused by scrapie [10,11]. Such studies have provided invaluable materials for the development of immunochemical and immunohistochemical discriminatory tests that have the ability to differentiate between BSE and scrapie [12-17].

Application of these discriminatory tests in national surveillance schemes is now a statutory requirement, and has revealed a caprine BSE case in France [18]. Retrospectively, another caprine TSE case was identified and confirmed as BSE in the UK [19,20]. Part of this statutory process is the confirmation of the suspicion of BSE by mouse bioassay.

Although the discriminatory tests have been validated rigorously on the basis that a small ruminant may be affected by either BSE or scrapie, their performance on samples where BSE and scrapie prions concur has not been tested despite evidence that co-infection by multiple TSE strains in a single animal is plausible [21-23]. Therefore the possibility that sheep in commercial holdings might have been affected by BSE from feed, which subsequently remains undetected due to the simultaneous presence of scrapie, cannot be excluded unequivocally. In contrast to the pathogenesis of BSE in cattle where the PrP^Sc^ distribution is predominantly detectable in the central nervous system, except for a short period after oral challenge where it can be identified in the Peyer’s patches and in the myenteric plexus of the distal ileum [24,25], the peripheral pathogenesis of BSE in sheep is similar to that of scrapie with extensive accumulation of the infectious agent in peripheral tissues [26,27]. This widespread distribution of the infectious agent is considered to be the main factor responsible for sheep-to-sheep transmission of scrapie [28] and similarly BSE infectivity in a flock could reasonably be sustained even after the original source of infection is eliminated. Recent evidence suggesting that natural transmission of BSE in sheep can occur [29] seems to confirm this hypothesis. This possibility warrants careful consideration, since the widespread peripheral distribution of BSE prions in sheep suggests that measures that were responsible for the successful eradication of BSE from the cattle population would appear inadequate to eliminate BSE in sheep. Indeed this makes it virtually impossible to ensure absence of BSE infectivity to consumers after consumption of sheep meat or its products [27].

It is evident that there is a clear need to detect BSE in small ruminants not just when it is the only infectious prion present but also when it may co-exist with scrapie, possibly in the same animal. Under such conditions discrimination of BSE from scrapie may not be straightforward since, according to the only experimental co-infection study where mice were challenged with mixtures of mouse adapted BSE and scrapie, only the scrapie phenotype was recognised based on differing electrophoretic properties of PrP^Sc^ [30]. This suggests that scrapie masked the BSE signal in the original inoculum and that following transmission to mice scrapie either propagated at the expense of BSE or the scrapie phenotype masked any signal that might had been generated by simultaneous propagation of the BSE agent.

Despite the potentially high exposure of small ruminants to BSE-contaminated feeds only a small proportion of BSE cases were identified throughout Europe, both of them in goats. No BSE has been currently detected in sheep worldwide. This has led to the belief that if BSE had entered in the sheep population the infectivity levels would be low, and in such cases it could escape diagnosis if it resided in sheep which were also infected with a high scrapie titre. In this study we investigated the ability of wild type mouse bioassay to identify BSE when it is present in an excess of scrapie during primary isolation using histopathology, immunohistochemistry (IHC) and Western blot.

## Results

### Attack rate (AR), hit rate (HR) and incubation period (IP) analysis

The AR and HR of all isolates are presented in Table 1. AR is defined as the ratio of TSE positive animals over the total number of challenged animals. HR is defined as the ratio of TSE positive animals over the number of animals encountered after the first TSE positive animal. In our view, AR is considered to be a better indicator of transmissibility than HR when species barriers have to be overcome, particularly when they have an adverse effect on transmissibility. Therefore, for the remainder of the paper only AR will be considered although HR data are also presented for comparative purposes.

**Table 1:** **Attack rate (AR), hit rate (HR) and incubation period (IP) of scrapie/BSE mixtures inoculated in three wild type mouse lines**

**Mouse line**	**Bioassay parameters**	**Inoculum**					
		**100% scrapie**	**1% BSE**	**2% BSE**	**10% BSE**	**50% BSE**	**100% BSE**
RIII	AR	0.95	0.95	0.80	0.95	0.80	0.80
	HR	1.00	1.00	1.00	1.00	1.00	0.94
	IP	459 ± 17	457 ± 17	461 ± 17	458 ± 15	473 ± 17	424 ± 52
C57	AR	0.90	0.95	0.85	0.90	0.95	0.75
	HR	1.00	1.00	1.00	1.00	1.00	0.88
	IP	464 ± 34	481 ± 22	448 ± 48	485 ± 24	491 ± 36	595 ± 77
VM	AR	0.90	1.00	1.00	0.95	1.00	1.00
	HR	1.00	1.00	1.00	1.00	1.00	1.00
	IP	579 ± 86	635 ± 85	635 ± 62	666 ± 61	629 ± 100	638 ± 64

AR (Attack Rate) is the ratio of confirmed TSE positive mice over the number of inoculated mice (n = 20).

HR (Hit Rate) is the ratio of TSE positive mice over the number of animals encountered after the first positive clinical animal.

IP (Incubation Period) was recorded as days post inoculation and values indicate mean ± standard deviation. Only clinically and TSE confirmed positive mice were included in the IP assessment.

**Figure 1:**
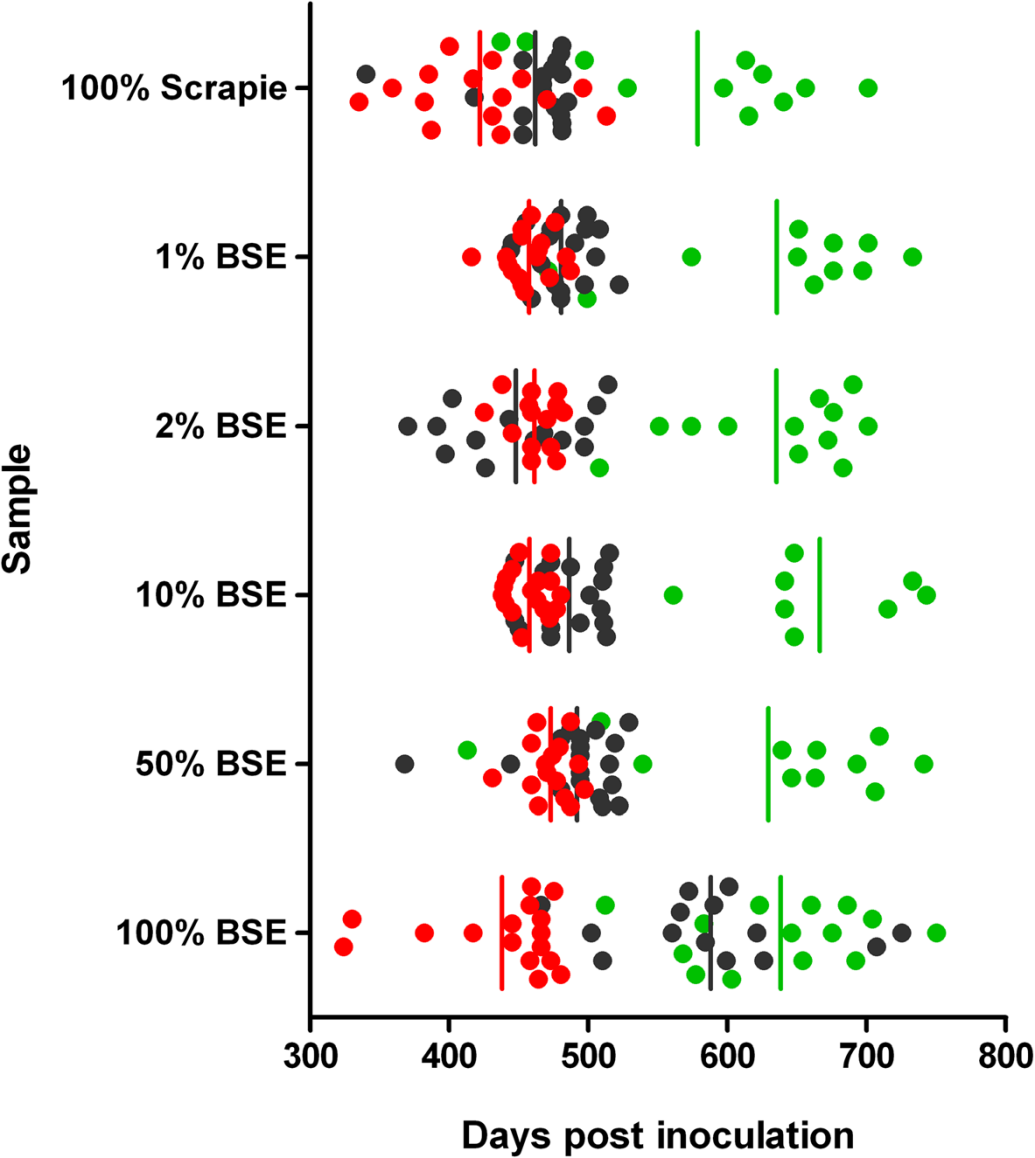
**Incubation period data of RIII, C57BL/6 and VM mice.** Animals were inoculated with various BSE/scrapie mixtures and the BSE and scrapie sources that were used to produce the mixtures. In the mixtures only the percentage of BSE is indicated; the remaining reflects scrapie percentage. Circles indicate individual mice, vertical lines indicate the mean of the group. RIII, red; C57BL/6, black; VM, green.

The IP data are expressed as days post inoculation (dpi) and are based on TSE positive mice that exhibited clinical signs of neurological disease (Table 1 and Figure 1). Generally there is too big an overlap between all inocula to allow any discrimination even between the controls, 100% scrapie and 100% BSE. The only notable observation was the prolonged IP of the 100% BSE source in C57BL/6 mice compared to all other isolates. The variance of IP in this group of mice was also increased when compared with the other C57BL/6 groups. Another, subtle difference was that in VM mice the mean IP of the 100% scrapie source was shorter than 600 dpi whilst the 100% BSE source and all the mixtures produced IP longer than 600 dpi. However this difference was not statistically significant (P = 0.0913, Log-rank (Mantel-Cox) test).

Both 100% scrapie and 100% BSE generated high AR. Compared to other inocula of the same TSEs prepared from terminally ill animals and bioassayed in our laboratory the IP values observed in this study were similar to the lower end of the IP data range indicating that that each source represented an origin of high infectivity [16,31].

### Lesion profile (LP) analysis

**Figure 2:**
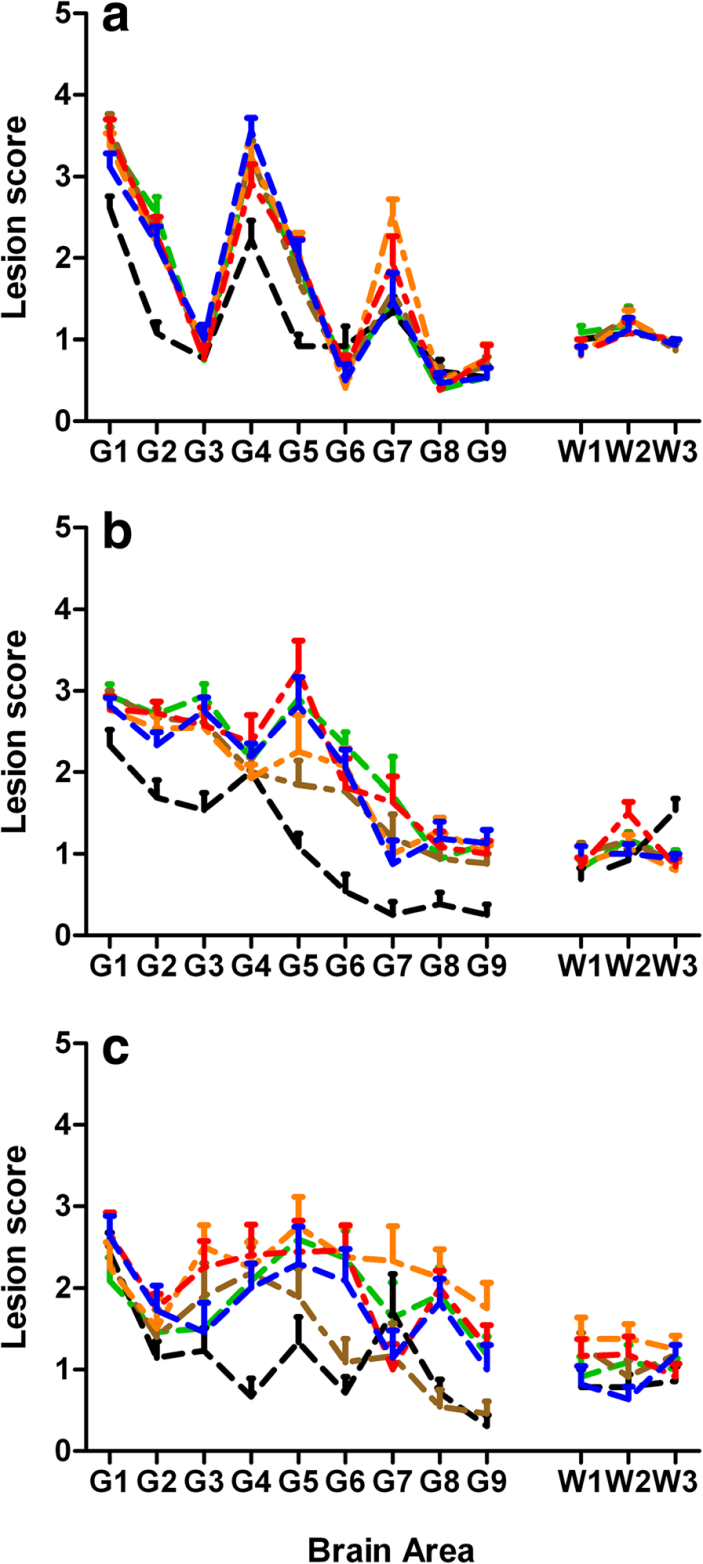
**Lesion profiles from RIII, C57BL/6 and VM mice.** RIII **(a)**, C57BL/6 **(b)** and VM **(c)** mice were inoculated with various BSE/scrapie mixtures and the BSE and scrapie sources that were used to produce the mixtures. BSE/scrapie mixtures are indicated by blue (1%BSE), red (2%BSE), amber (10%BSE) and brown (50%) lines; scrapie control, green line; BSE control, black line.

Due to the high infectivity of all inocula there were at least five clinically and pathologically positive mice in each mouse group. Therefore it was feasible to construct LP from each mouse group (Figure 2).

In the RIII mice the LP from mixtures appear to be more akin to the LP of the 100% scrapie source (Figure 2a). However, both the 100% BSE and 100% scrapie produced LP which showed similar contours, the main differences between the two being quantitative. This property of the RIII mice has been reported elsewhere and the scrapie associated profile has been designated 1-4-7-scrapie to denote its similarity to BSE induced LP [16] which also produces consistent LP with peaks in the same brain areas [32]. The 1-4-7-scrapie LP has only been derived from ARQ/ARQ scrapie sources [16,31]. In the current study the 100% scrapie source consisted mainly of VRQ/VRQ (45.16%) and ARQ/VRQ (9.68%) sources whist the ARQ/ARQ content was 40.32%.

The LP differences between the 100% BSE and 100% scrapie sources were more profound in C57BL/6 and VM mice, where they resulted in different LPs. The LPs that were generated from the various mixtures in these two mouse lines aligned more closely to the LP from the 100% scrapie control than the 100% BSE isolate.

In addition application of cluster analysis based on the lesion scores, showed that in all three mouse lines the BSE/scrapie mixtures grouped together with the 100% scrapie control which was distinct from the 100% BSE control (Additional file 1: Figure S1).

### Estimation of PrP^Sc^ levels in the source BSE and scrapie inocula

**Figure 3:**
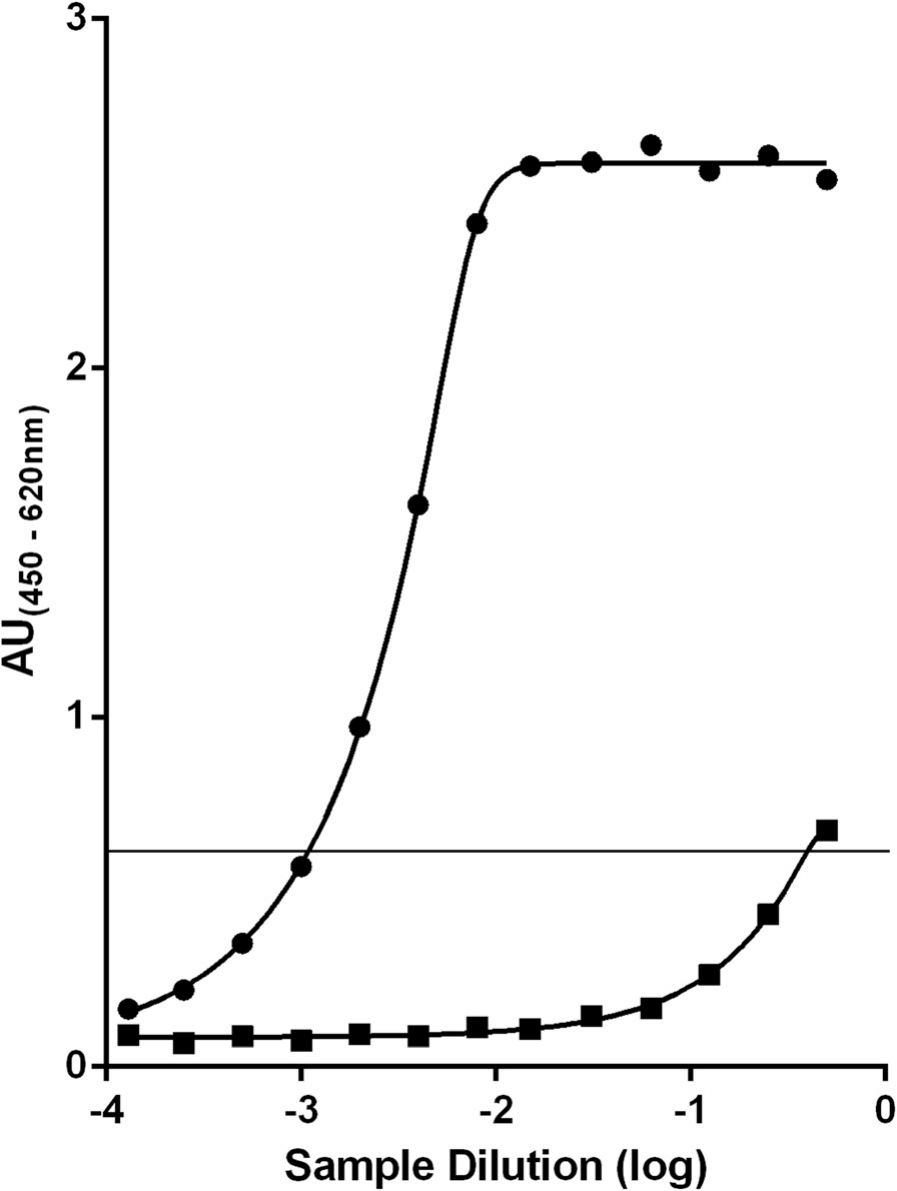
**Comparison of PrP** ^**Sc**^ **levels in BSE and scrapie inocula as determined by IDEXX EIA.** Both inocula, 100% BSE (■) and 100% scrapie (●), were analysed by IDEXX Herdchek BSE/scrapie EIA as described in Materials and methods. Colour development, measured in absorbance units (AU), is proportional to the amount of PrPSc in the sample when the growth phase of the curve is linear. Required inocula dilutions to achieve an AU value of 0.6, where the growth phase of the 100% BSE curve was most linear, were 1/912 for 100% scrapie and 1/2.5 for 100% BSE, indicating the quantity of PrP^Sc^ in the scrapie inocula was 357 times greater than in the BSE inocula. OD values of ovine BSE and ovine scrapie positive controls were 2.659 and 2.664 AU respectively. OD values of two control negative sheep samples were 0.064 and 0.057 AU.

The PrP^Sc^ levels in the 100% BSE and 100% scrapie homogenates were estimated using IDEXX Herdchek BSE – scrapie EIA kit, an ELISA-based commercially available TSE diagnostic method. Based on this approach comparison between BSE and scrapie sources indicated that the PrP^Sc^ levels in the 100% Scrapie homogenate were 2.6 logs higher compared to the 100% BSE inoculum (Figure 3).

### Western blot analysis

**Figure 4:**
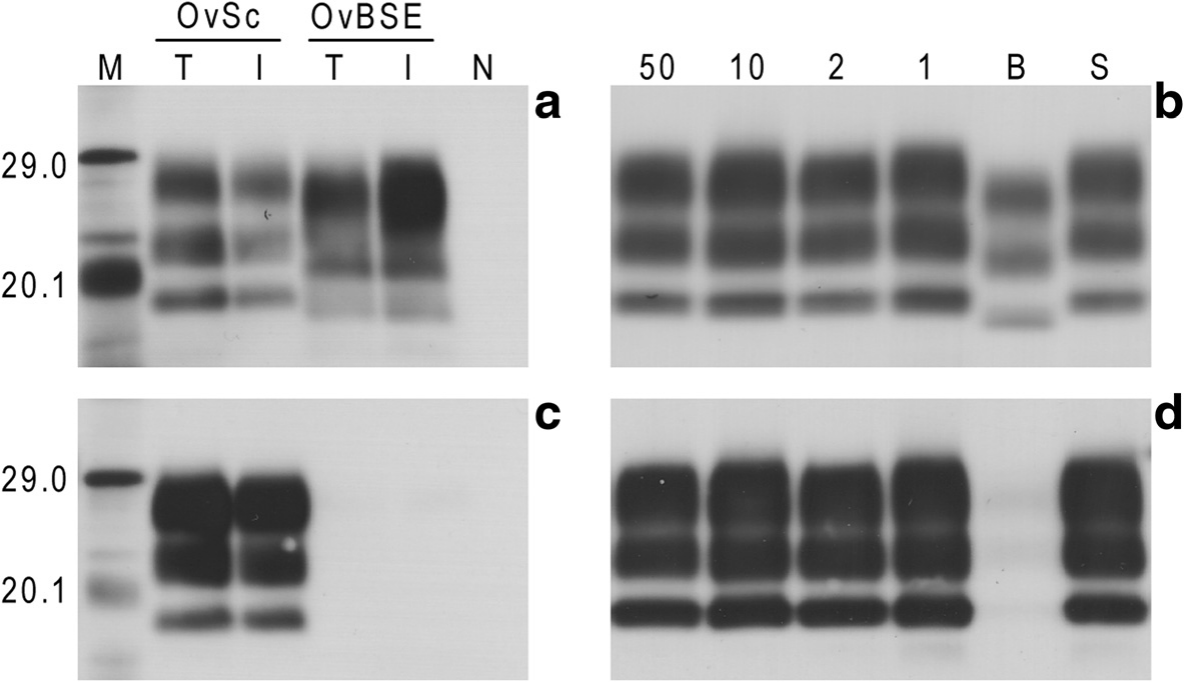
**Western blot analysis of inocula homogenates that were used to challenge the mice.** Western blots were performed as described in Materials and methods using antibodies Sha31 **(a and b)** and 12B2 **(c and d)**. Images a and c compare the western blot profiles obtained for the two different methods used to extract samples; solid tissues (T) and inocula (I). Images b and d show western blot profiles observed for the different prepared inolcula: 100% BSE (B), 50% BSE (50), 10% BSE (10), 2% BSE (2), 1% BSE (1) and 100% scrapie (S). For BSE/scrapie mixtures only the percentage BSE is indicated; the remainder indicates the percentage of scrapie. All inocula represent 10% (w/v) brain tissue in normal saline. An inoculum prepared from a TSE negative sheep was also analysed by Western blot (N). Molecular mass markers (M) are indicated in kDa. For **(a)** and **(c)** each sample was diluted to achieve optimum representation of banding profile; dilution of each source between **(a)** and **(c)** was kept constant. For **(b)** and **(d)** each lane was loaded with 15 μl of neat extracted sample, equivalent to 30 mg of brain tissue.

Application of Western blot to the inocula that were used to challenge the mice showed that all the mixtures essentially behaved as scrapie, showing a higher molecular weight unglycosylated band compared to BSE (Figure 4a) and a strong binding affinity for antibody 12B2 (Figure 4b).

**Figure 5:**
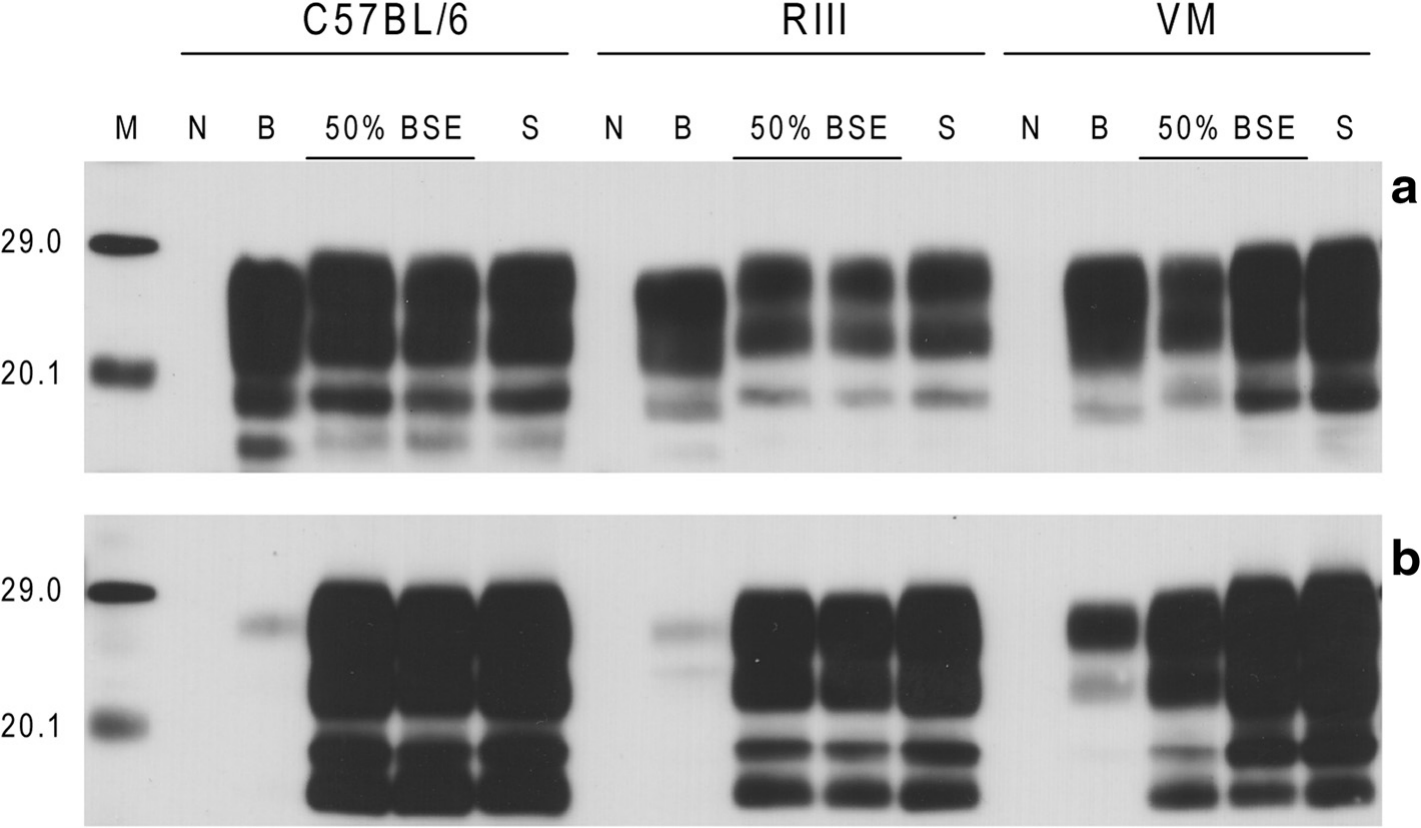
**Western blot analysis of mice inoculated with BSE/scrapie mixtures.** Western blots were performed as described in Materials and methods using antibodies Sha31 **(a)** and 12B2 **(b)**. Western blots show three mouse lines (C57BL/6, RIII & VM) challenged with 100% BSE (B), 50% BSE (50% BSE) and 100% scrapie (S). Brain tissue from an unchallenged mouse (N) was also analysed. Molecular mass markers (M) are indicated in kDa. All samples were extracted from fresh frozen tissue. Each lane was loaded with 15 μl of neat extracted sample, equivalent to 30 mg of brain tissue.

Application of Western blot to the brains of C57BL/6 and RIII mice showed that even animals that were challenged with the highest BSE/scrapie ratio (50% BSE) were indistinguishable from those that were challenged with the scrapie control, showing a high molecular weight unglycosylated band compared to the BSE control (Figure 5). A fourth band running approximately 2 kDa below the unglycosylated band was observed in all murine scrapie samples. This, otherwise faint, band became prominent when the gels were visualised using antibody 12B2. This band is thought to represent C-terminal cleavage products of PrPSc. Mice inoculated with BSE/scrapie mixtures where the relative proportion of BSE was less than 50% also showed a Western blot phenotype that was indistinguishable from that induced by scrapie (data not shown). No attempt was made to apply Western blot to VM mice as in this mouse line BSE cannot be distinguished from scrapie using this technique [33].

### Immunohistochemical (IHC) analysis

Slides were divided into three groups, according to the three mouse lines used: C57BL/6, RIII and VM.

All slides were interpreted by a single assessor. During the first phase, the 100% BSE and 100% scrapie samples were evaluated unblinded, for the reader to identify the hallmarks of the PrP^Sc^ patterns associated with each agent [34-36]. The remaining slides, which derived from mice inoculated with 1%, 2%, 20% and 50% BSE, were all mixed randomly and potential IHC markers assessed blind by the reader (Phase 1).

**Figure 6:**
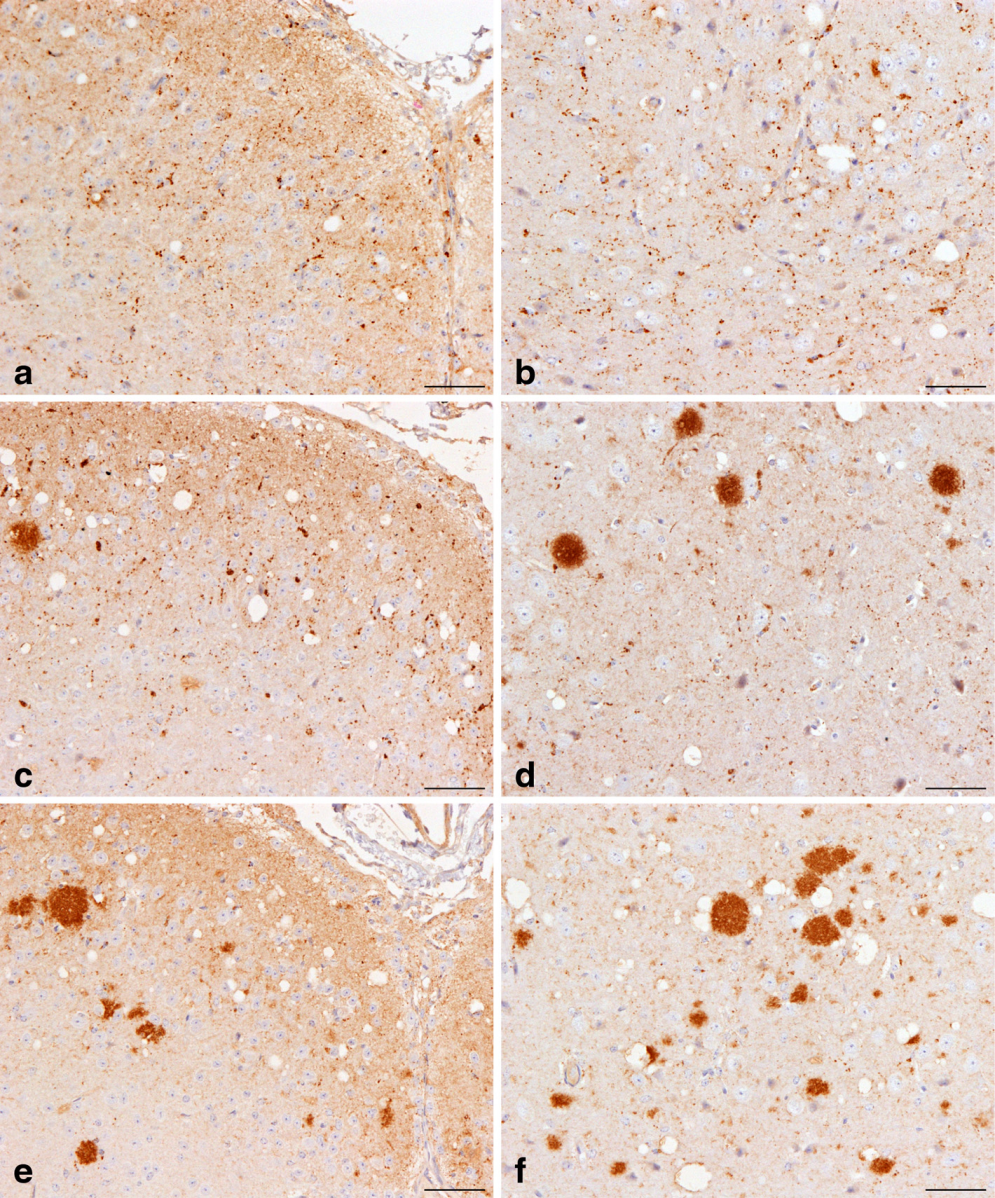
**Detection of PrP** ^**Sc**^ **types using immunohistochemistry in mouse brains.** Mice challenged with BSE show BSE-characteristic punctuate PrP^Sc^ deposits **(a, b)** which are the only evident BSE feature in mice challenged with BSE/scrapie mixtures **(c, d)** in a pattern that is otherwise indistinguishable from scrapie **(e, f)**. The content of BSE in the BSE/scrapie mixture used to inoculate the mice shown in **c** and **d** was 50%; **a**, **c**, **e** superior coliculus; **b**, **d**, **f** thalamus. Scale bar represents 50 μm. PrP^Sc^ was detected with a polyclonal rabbit antibody Rb486 diluted 1/2000.

**Figure 7:**
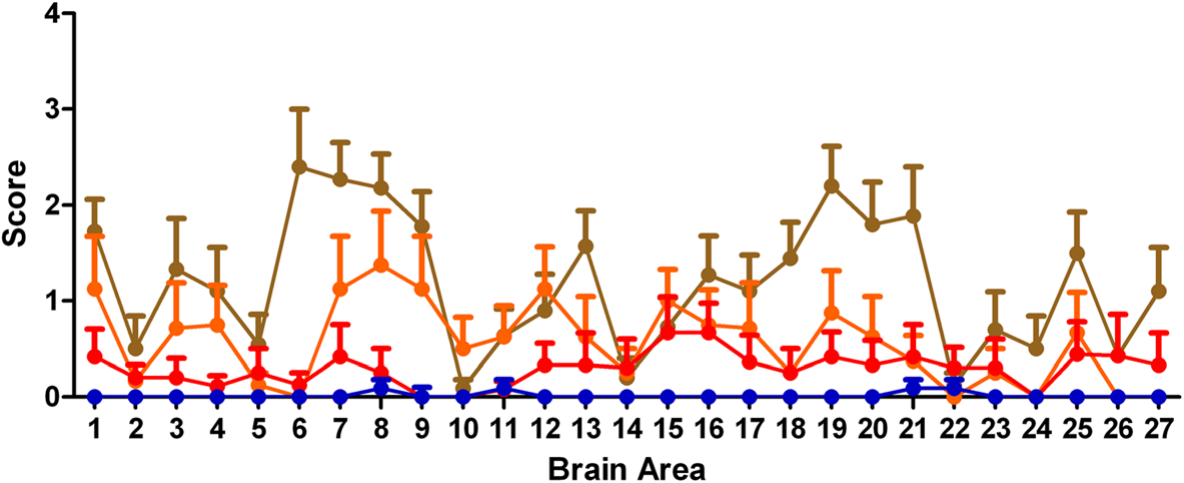
**Assessment of intensity and distribution of BSE**-**associated punctate PrP** ^**Sc**^ **deposits in the brain.** The amount of BSE-associated punctate PrP^Sc^ deposits was were assessed semiquantitatively from 0–4 in 27 neuroanatomical areas of clinically positive VM mice challenged with BSE/scrapie mixtures and the scores were plotted against the areas. The brain areas scored were: 1, medulla; 2, locus coeruleus; 3 cerebellar pedunculi; 4, cochlear nuclei; 5, dorsal cerebellum (granular layer); 6, inferior colliculus; 7, superior colliculus; 8, white matter in the mesencephalic tegmentum; 9, medial geniculate nuclei; 10, peri-acquaductal grey matter; 11, Raphe nuclei; 12, red nuclei; 13, substantia nigra; 14, interpeduncular nucleus; 15, hypothalamus; 16, lateral hypothalamus; 17, zona incerta; 18, anterior dorsal thalamic nuclei; 19, laterodorsal thalamic nuclei; 20, lateral thalamus; 21, thalamus; 22, habenular bodies; 23, hippocampus; 24, septal nuclei of the paraterminal body; 25, nucleus of the vertical limb of the diagonal band; 26, medial preoptic band; 27, ventral pallidum. The content of BSE in the BSE/scrapie mixtures was 50% (brown line), 10% (amber line), 2% (red line) and 1% (blue line). Error bars indicate SEM.

**Figure 8:**
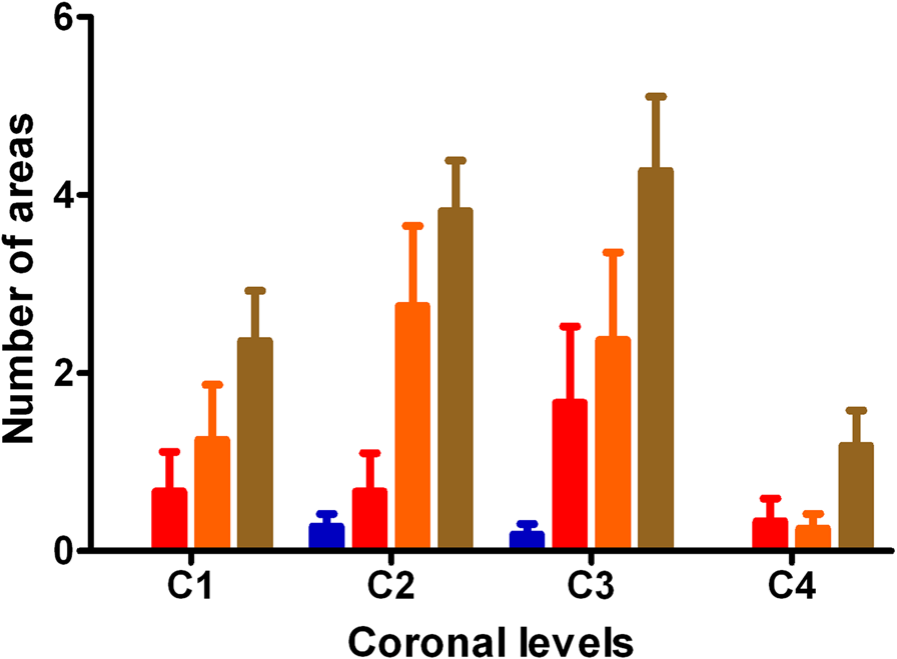
**The number of neuroantomical areas affected by BSE-** **associated PrP** ^**Sc**^ **deposits at four different coronal levels.** C1, C2, C3 and C4 correspond to medulla, midbrain, thalamic and frontal coronal levels respectively. These four coronal levels (C1-C4) are routinely used in TSE strain typing studies. The content of BSE in the BSE/scrapie mixtures was 1% (blue columns), 2% (red columns), 10% (amber columns) and 50% (brown columns). Error bars indicate SEM.

At the end of Phase 1 it was concluded that it was not possible to recognise any BSE associated markers in either C57BL/6 or RIII mice challenged with BSE/scrapie mixtures as all animals showed a single phenotype that was indistinguishable from the phenotype induced by the 100% scrapie control. In VM mice challenged with BSE/scrapie mixtures, however, a single PrP^Sc^ type which was initially observed in VM mice challenged with BSE but not with scrapie, designated ‘BSE-associated punctate pattern’, was identified (Figure 6). This PrP^Sc^ type appeared to be present in a number of BSE/scrapie challenged mice that otherwise had a scrapie compatible IHC phenotype. Subsequently, this BSE specific PrP^Sc^ type was assessed semi-quantitatively on a scale 0 to 4 (0 = none, 1 = inconclusive, 2 = mild, 3 = moderate and 4 = abundant) in different neuroanatomical areas, located at four coronal levels (medulla, midbrain, thalamic and frontal) in all BSE/scrapie challenged mice. This assessment, which was performed blind, revealed that this pattern was more abundant in mice that were challenged with mixtures with a higher BSE concentration, both in terms of quantity in the different neuroanatomical areas (Figure 7) and its distribution throughout the brain (Figure 8).

Based on the previous finding that the BSE-associated punctate PrP^Sc^ type can be observed in BSE/scrapie mixtures, all samples derived from VM mice challenged not only with BSE/scrapie mixtures but also with 100% BSE or 100% scrapie were mixed randomly and assessed blind by the same operator (Phase 2). The presence of BSE-associated punctate deposits in specific neuroanatomical areas allocated in the four coronal levels was recorded. At each coronal level the number of neuroanatomical areas affected by BSE-associated punctate deposits was used to predict the presence and percentage of BSE in the original inoculum. The test predictions were compared to the actual values and the associations between test and true status were not random (p < 0.0001 by Fisher’s exact test). The sensitivity, specificity and accuracy of the approach were 78% (62–89), 91% (75–98) and 84% (73–91); values in brackets denote 95% confidence intervals. The predictions were more accurate in the high BSE content inocula (50% and 10%) whilst the low BSE content mixtures (2% and 1%) proved more difficult to predict accurately.

## Discussion

Despite the significant progress and development of tests that can discriminate BSE from scrapie in a single infection scenario, limited studies have been conducted that address the performance of the discriminatory tests in cases where BSE and scrapie exist in the same animal. Ideally, the best possible experimental materials should derive from sheep co-infected experimentally with natural scrapie and BSE sources via natural routes of inoculation i.e. under conditions that would reflect most closely a naturally occurring co-infection. To the best of our knowledge, however, materials from such experiments are not widely available. Therefore we used the best possible alternative i.e. we produced BSE and scrapie mixtures *in vitro* and used them as a substitute to co-infected tissue. Both BSE and scrapie inocula were prepared from animals at the terminal stage of the disease to ensure maximum infectivity. However, measurement of PrP^Sc^ levels indicated that the concentration of PrP^Sc^ in the scrapie source was at least two logs higher compared to the BSE material. Conversely, it has been shown the TSE infectivity cannot be accurately predicted from quantitative laboratory test results [37]. A similar approach has been followed in another study where it was attempted to distinguish BSE from scrapie under co-infection conditions using wild type mice [30]. In that report the BSE and scrapie sources were mouse adapted sources whilst in the current study we used ovine tissues to replicate closer a scenario where potentially co-infected ruminant tissues may be used to challenge rodents.

Based on AR, IP and LP our data suggest that all three mouse lines that were challenged with BSE/scrapie mixtures exhibited a scrapie phenotype, and it was not possible to identify any BSE attributes in the mixtures. However, AR, IP and LP data are considered to be less reliable parameters with lower discriminatory power since they are based on average values derived from a group of animals [16,38].

Each mouse that was challenged with either a BSE/scrapie mixture or 100% scrapie control in the current study, exhibited a stable scrapie IHC pattern which is usually isolated from ARQ/ARQ scrapie cases [16,31,38] although these sources comprise 40.32% of the scrapie pool. In C57BL/6 or RIII mice this IHC pattern was indistinguishable from that observed in mice challenged with 87A [34,38].

The Western blots from C57BL/6 and RIII mice that were challenged with BSE/scrapie mixtures showed that even with the highest BSE fraction (50%) the scrapie characteristics dominated and any BSE signal was undetectable within the resolution limits of this method. Using IHC it was not feasible to identify any BSE characteristics in either C57BL/6 or RIII mice that were challenged with BSE/scrapie mixtures so there is complete agreement between IHC and Western blot data regarding these two mouse lines. The difference in the PrP^Sc^ levels between the scrapie and BSE sources could be a possible explanation of this result.

Collectively the above data indicate that in the C57BL/6 and RIII mice that were challenged with BSE/scrapie mixtures only scrapie phenotypic traits were identified, suggesting that either scrapie propagated preferentially at the expense of BSE or, that although BSE also propagated, it had a recessive phenotype that was not observed in the mice. The similarity of results obtained from C57BL/6 and RIII mice is likely explained by the fact that these two mouse lines share the same PrP sequence (Prnp-a), that influences the phenotypic features of TSE strains [38-40].

Propagation of a mixture of strains is supported by the data from the VM mice where, while the overall IHC characteristics of scrapie prevailed, a subtle but distinct BSE feature was observed in mice challenged with BSE/scrapie mixtures, and that feature was more prominent in the mice that were exposed to high content of BSE (10% or 50%) compared to those that received a low content of BSE (1% or 2%). Although we were able to identify a BSE associated trait that could potentially be used to detect BSE in the presence of scrapie using VM mice, the current study shows that overall scrapie can dominate the phenotype of the disease even if BSE prions propagate in the background, while some phenotypic aspects of BSE may be evident. This disparity in the properties of different mouse lines could be attributed to the different PrP sequences between VM and C57BL/6 or RIII mice. However, the VM data show evidence that BSE and scrapie can co-exist in the same animal, although the presence of BSE may be masked by a dominant scrapie phenotype.

Great care must be taken, however, when attempting to make any generalizations from this data. Firstly the infectious titre of each of the two sources that were used to produce the mixtures was not evaluated therefore the mixtures did not reflect titre ratios but simply volumetric fractions. Although, according to some researchers, it would have been preferable to use titrated material this would have prolonged the length of the study by at least another two years. In addition, titres are the function of the strain/ host combination used (BSE is usually titrated in RIII mice whilst scrapie is usually titrated in C57BL/6 mice) and cross reading between different strain/host systems is not always informative or appropriate. Therefore it is questionable whether titre matching provides an optimal approach to co-infection experiments. It could also be argued that the mixtures could have been based on PrP^Sc^ concentration. However, the content of PrP^Sc^ in an inoculum may not always correlate directly with infectivity [37]. Further evidence that this may be the case is provided by reports that although PrP^Sc^ levels in ovine BSE can be lower compared to cattle BSE infectivity titres between the two samples can be similar [41-43]. Nevertheless, in the current study both sources generated presented fairly high AR and short IP compared to similar inocula that have been previously bioassayed in our institute in all three wild type mouse lines used indicating that both had relatively high infectivity [31,32]. The interactions between different scrapie strains and BSE is another parameter that must be considered. In this study all mice showed IHC patterns that were associated with scrapie strains that are related to the ARQ/ARQ genotype, 87A in Prnp-a mice and 87 V in Prnp-b mice [31,38,44]. It is not possible to predict how other scrapie strains, such as ME7 or 221C, would interact with BSE.

Since the initiation of the current study transgenic mouse lines which are more sensitive to specific animal TSEs have been introduced and validated. Such lines are relatively new, but promising, as intermediate phenotypes have been identified after challenge with BSE/scrapie mixtures prepared *in vitro* [45,46]. However, these preliminary data require further validation to evaluate the ability of these models to detect BSE in the presence of scrapie. Recently, a serial protein misfolding cyclic amplification assay (sPMCA) has been developed to specifically detect classic BSE even within the presence of scrapie prions. It has been reported that in a blind trial, this sPMCA-based assay specifically amplified BSE PrP^Sc^ within brain mixes with 100% specificity and 97% sensitivity when BSE agent was diluted into scrapie-infected brain homogenates at 1% (vol/vol) [47]. Compared to mouse bioassay this in vitro technique may offer a quick, reliable and more cost effective method in detecting BSE in the presence of scrapie.

The fact that we were able to identify a marker to detect BSE against a specific scrapie strain background in VM mice does not mean that this is an acceptable test for BSE detection in co-infected material. It does, however, give further understanding of the potential and limitations of such an approach, which is relevant to the retrospective interpretation of historical data in this field, in particular to prevent the over-interpretation of ‘absence of BSE’ conclusions. It must be noted that the selected marker is subtle and only an experienced observer could interpret it correctly and consistently in the high content BSE inocula (10% or 50%), as in the low content BSE mixtures (1% and 2%) the levels of the marker dropped appreciably. Therefore, even using this IHC model, low BSE levels on a scrapie background may remain undetected. Theoretically similar caveats may apply to the natural host ie small ruminants, where there is a range of strain and host genotype combinations. Consequently, the diagnostic methodology applied in surveillance schemes may fail to identify co-infection cases where BSE is present. In the view of the authors, under these circumstances, the current policy of eradicating or minimizing scrapie rather than attempting to detect and manage BSE separately remains, scientifically and financially, the most feasible option to prevent BSE from entering the food chain via small ruminants.

## Conclusions

In conclusion, our data suggest that by applying immunohistochemistry to detect different PrPSc types in the brains of mice challenged with BSE/scrapie mixtures it is possible to detect BSE in a BSE/Scrapie co-infection scenario. We also provide evidence that in principle co-infection of BSE and scrapie cannot be excluded at least in an experimental model.

## Materials and methods

### Ethical statement

All animal procedures were performed in compliance with the Animal (Experimental Procedures) Act 1986 under license issued by the UK Home Office (license number PPL70/5155), and were approved by the local ethics committee.

### Inoculum preparation

Initially a scrapie and an ovine BSE homogenate were prepared. The scrapie inoculum, hereafter referred to as 100% scrapie, was made using a pool of brains collected between 1996 and 1999 from confirmed cases of natural scrapie representing the most frequent PrP genotypes affected by scrapie (Table 2). The ovine BSE inoculum, hereafter referred to as 100% BSE, was prepared from a pool of ARQ/ARQ sheep brains which had been challenged experimentally with bovine BSE and subsequently succumbed to clinical disease. The brains were homogenised using a handheld blender and made into a 10% suspension in normal saline. These 10% suspensions were filtered through a single layer of sterile gauze to remove large pieces of tissue, tested for microbiological sterility and treated with antibiotics if necessary. Aliquots of these two inocula were combined to produce mixtures of BSE and scrapie at the following relative ratios: 1% BSE :99% scrapie (hereafter referred to as 1% BSE), 2% BSE :98% scrapie (hereafter referred to as 2% BSE), 10% BSE:90% scrapie (hereafter referred to as 10% BSE) and 50% BSE:50% scrapie (hereafter referred to as 50% BSE). All homogenates were stored at −80°C until they were used for inoculations.

**Table 2:** **Genotype constitution of the 100% scrapie pool**

	**Animals**	
**Genotype**	**Number**	**%**
ARQ/ARQ	25	40.32
ARQ/VRQ	6	9.68
VRQ/VRQ	28	45.16
ARR/VRQ	1	1.61
AHQ/ARQ	1	1.61
ARH/ARQ	1	1.61
Total	62	100

### Animal procedures

Each inoculum was administered into three panels of wild type mice namely C57BL/6, RIII and VM. Each panel consisted of 20 mice and each mouse received 20 μl of homogenate intracerebrally and 100 μl intraperitonially. Intracerebral inoculations were performed under general anaesthesia using a 25 G hypodermic needle attached to a 0.5 ml insulin syringe. A plastic sheath was inserted along the needle allowing approximately 5 mm of free end of the needle to be exposed to ensure that the inoculum was deposited at similar depth in each animal and to minimize tissue injury. The point of entry was approximately 3 mm dorsal to a point lying halfway between the eye and the base of the ear. Insertion into the CNS was achieved by gently rotating the syringe along its axis at a right angle with respect to the skull surface.

Mice were housed in standard mouse cages and were monitored for clinical signs of disease by experienced animal attendants. Mice were euthanized after exhibiting clinical signs of TSE for two consecutive weeks or having received scores of “definitely affected” in 2 out of 3 consecutive weeks unless the clinical progression of the disease was so rapid that animals had to be euthanized on welfare grounds. Mice that did not show clinical signs of TSE were allowed to live until they were euthanized on welfare grounds due to other conditions (intercurrent deaths).

At postmortem the brain of each mouse was removed sectioned along a parasagittal plane into two parts. One third of the brain was stored at −80°C for further bioassay or biochemical studies and two thirds were immersed in buffered formalin for 72 hours at room temperature.

After fixation was completed each brain was cut at 4 different coronal points at the level of medulla (including the cerebellum), midbrain, thalamus (including hippocampus and overlying cortex) and frontal cortex (including basal ganglia). Each coronal segment was embedded in paraffin wax and histological sections (3 μm thick) from each level were mounted on the same positively charged slide for interpretation.

### Histopathological assessment

Slides were stained with haematoxylin and eosin according to standard methodology [48]. TSE diagnosis was based on the presence of TSE specific vacuolation and each sample was diagnosed as TSE positive, negative or inconclusive. On TSE positive samples the lesion intensity in specific neuroanatomical areas was further assessed semi-quantitatively and assigned a score on a scale 0–5 (grey matter areas) or 0–3 (white matter areas). The lesion scores from clinically and histopathologcally positive samples were plotted against the respective brain areas to produce lesion profiles as described previously [32,49].

### Enzyme immunoassay

Inocula samples were analysed using a modified version of the IDEXX Herdchek BSE – Scrapie EIA kit (IDEXX laboratories, One IDEXX Drive, Westbrook, Maine 04092, USA). Inocula samples were serially diluted in equal volumes of TSE negative ovine brain homogenate (10% (w/v)) prepared in IDEXX kit homogenisation buffer. Diluted samples were treated as if they were normal test homogenates (normally prepared as 25% (w/v)) and assayed following manufacturer’s instructions. Briefly, diluted samples were mixed 4:5 with kit plate diluent, distributed (100 μl) to the test plate and incubated for 180 minutes at ambient room temperature (RT). After washing, bound sample was incubated with conditioning buffer for 10 minutes at RT, washed a second time and incubated with horseradish peroxidase conjugated anti-PrP antibody (SRB-CC) for 90 minutes at RT. After final wash visualisation of bound PrP^Sc^ was achieved using 3,3′,5,5′-tetramethylbenzidine (TMB) substrate for 20 minutes at RT and measuring colour development at 450 nm using a reference filter at 620 nm (Perkin Elmer Envision 2104 multi-label reader).

### Western blot

All samples, solid tissue and inocula, were analysed using the Bio-Rad TeSeE™ Western blot (Bio-Rad Laboratories Ltd., Hemel Hempstead, Hertfordshire, UK).

Sheep and mouse brain (solid) tissues were analysed according to manufacturer’s instructions. Briefly, 20% (w/v) tissue homogenates were treated with proteinase K before alcohol precipitation. After centrifugation, pellets were solubilised in Laemmli buffer. Following extraction proteins were separated on 12% Bis/Tris gels then electrotransferred to PVDF membrane. After blocking the membrane with 5% bovine serum albumin (BSA) proteins were labelled with anti-prion antibodies Sha31 (epitope: ovine amino acid sequence 145–152) and 12B2 (epitope: ovine amino acid sequence 97–115). Visualisation was achieved using enhanced chemiluminesence (ECL) reagents (Amersham Biosciences; Little Chalfont, Buckinghamshire UK).

Prior to biochemical analysis of inoculum samples by Bio-Rad TeSeE™ Western blot, aliquots of finalised inocula were centrifuged at 350000 g for 30 minutes. The resulting pellet was re-homogenised at 20% (w/v) in Bio-Rad kit homogenisation buffer and the resultant homogenate treated as described above.

Prior to biochemical analysis of inoculum samples by Bio-Rad TeSeE™ Western blot, aliquots of finalised inocula were centrifuged at 350000 g for 30 minutes. The resulting pellet was re-homogenised at 20% (w/v) in Bio-Rad kit homogenisation buffer and the resultant homogenate treated as described above.

### Immunohistochemical labelling of PrP^Sc^

Samples from clinically and histopathologically positive mice were further analysed with IHC as described previously [16,31]. PrP^Sc^ was detected using the rabbit polyclonal anti-PrP antibody Rb486 according to standard methodology [48].

### Statistical analysis

Cluster analysis was performed using Statistica (Version 10). For all other statistical analyses the STATA (Version 10) or the Graph Pad Prism (Version 6.04) programmes were used.

## Additional file

Additional file 1: Figure S1. Cluster analysis of vacuolation score data. Vacuolation was assessed semiquantitatively in the same areas used for lesion profiling. The analysis shows that in all three mouse lines the BSE/scrapie mixtures group together with the scrapie control and this cluster is clearly separated from the BSE control.

## Abbreviations

AR: Attack rate(s)
AU: Absorbance units
BSA: Bovine serum albumin
BSE: Bovine spongiform encephalopathy
dpi: Days post inoculation
ECL: Enhanced chemiluminesence
EU: European Union
HR: Hit rate(s)
IHC: Immunohistochemistry
IP: Incubation period(s)
LP: Lesion profile(s)
OD: Optical density
PVDF: Polyvinylidine fluoride
TSE(s): Transmissible spongiform encephalopathy(ies)
UK: United Kingdom of Great Britain and Northern Ireland
vCJD: Variant Creutzveldt-Jakob disease
In PrP, Protein genotype abbreviations A: represents alanine, V valine, R arginine and Q glutamine
