# Supplementary figures and images for: Ability of wild type mouse bioassay to detect bovine spongiform encephalopathy (BSE) in the presence of excess scrapie
Source: Acta Neuropathol Commun. 2015 Apr 3;3:21. doi: 10.1186/s40478-015-0194-2 (PMC4382846; doi:10.1186/s40478-015-0194-2)

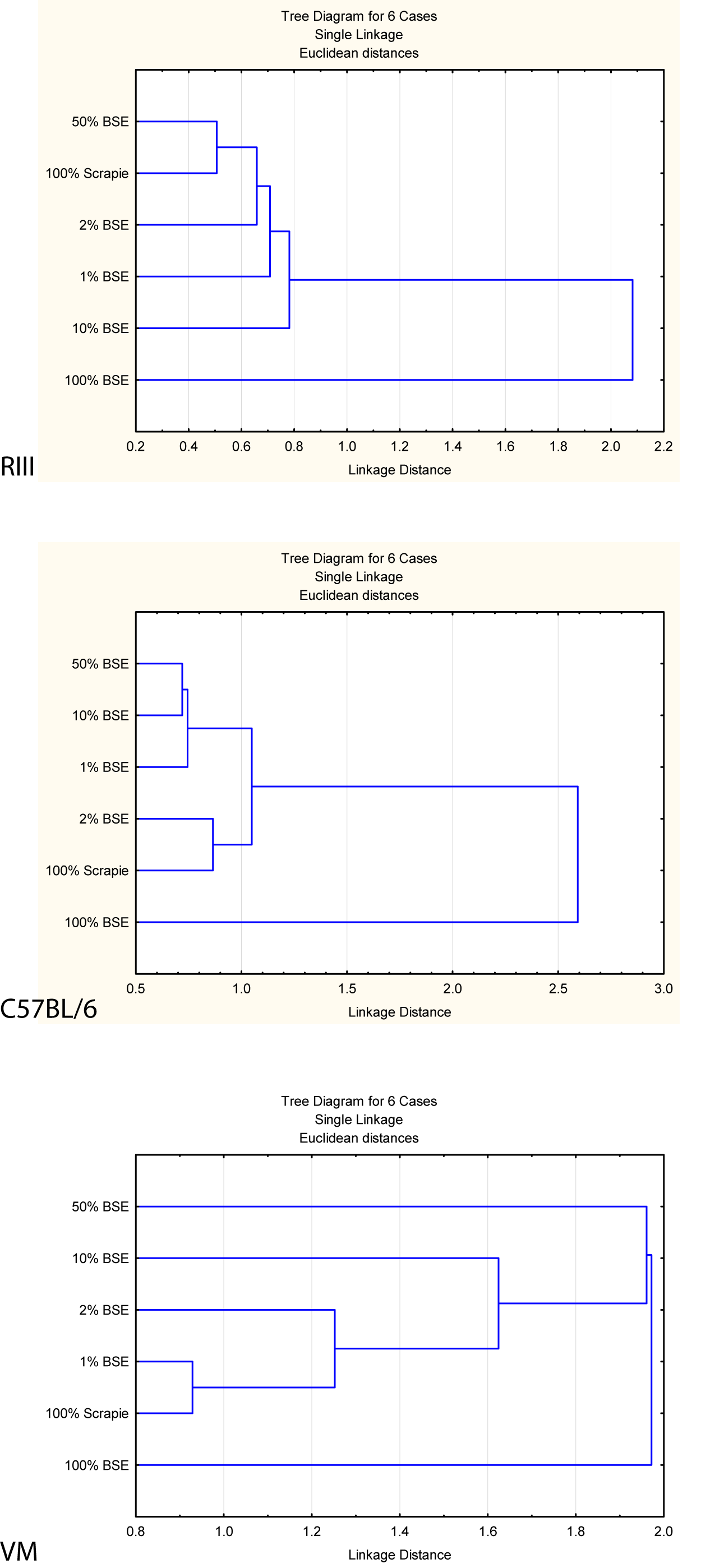

Supplement: Additional file 1: Figure S1. — Cluster analysis of vacuolation score data. Vacuolation was assessed semiquantitatively in the same areas used for lesion profiling. The analysis shows that in all three mouse lines the BSE/scrapie mixtures group together with the scrapie control and this cluster is clearly separated from the BSE control. [file 40478_2015_194_MOESM1_ESM.tif]
